# Actuator Fault Detection and Fault-Tolerant Control for Hexacopter

**DOI:** 10.3390/s19214721

**Published:** 2019-10-30

**Authors:** Ngoc Phi Nguyen, Nguyen Xuan Mung, Sung Kyung Hong

**Affiliations:** Department of Aerospace Engineering, Sejong University, Seoul 143-747, Korea; phinguyen.183@gmail.com (N.P.N.); xuanmung1009@gmail.com (N.X.M.)

**Keywords:** fault diagnosis, hexacopter, fault-tolerant control, sliding mode control, Thau observer

## Abstract

In this paper, fault detection and fault-tolerant control strategies are proposed to handle the issues of both actuator faults and disturbances in a hexacopter. A dynamic model of a hexacopter is first derived to develop a model-based fault detection system. Secondly, the altitude control based on a sliding mode and disturbance observer is presented to tackle the disturbance issue. Then, a nonlinear Thau observer is applied to estimate the states of a hexacopter and to generate the residuals. Using a fault detection unit, the motor failure is isolated to address the one or two actuator faults. Finally, experimental results are tested on a DJI F550 hexacopter platform and Pixhawk2 flight controller to verify the effectiveness of the proposed approach. Unlike previous studies, this work can integrate fault detection and fault-tolerant control design as a single unit. Moreover, the developed fault detection and fault-tolerant control method can handle up to two actuator failures in presence of disturbances.

## 1. Introduction

The multicopter unmanned aerial vehicles (UAVs) are drawing attention in the academic community. The have been developed and tested in several technologies, such as formation flight [1,2], precision landing [3,4], tracking control [5,6], remote sensing [7,8,9,10]. This significant growth has resulted from the following advantages that UAVs have, such as agility, economical cost, compactness, mechanical simplicity, and ability to operate in indoor and outdoor environments [11]. One of the control problems that a multicopter faces is actuator failure, which may cause many crashes and expose human beings to injury risks during operation [11]. Therefore, a fault-tolerant control (FTC) topic is a key factor in ensuring the safety and reliability demands of multicopters during flight missions. The goal of this paper is to propose a fault detection and fault-tolerant control method for hexacopters in the presence of one or two actuator failures.

### 1.1. Related Review

Studies on actuator fault detection (FD) and FTC for multicopter UAVs have been carried out intensively over the past decade. There are two types of failures in multicopter UAVs: partial loss and complete loss effectiveness in actuators. Several methods have been proposed to improve the stability and tracking performance for quadcopter UAVs in the presence of a partial actuator failure. Several control methods based on sliding mode control (SMC) [12,13,14] and adaptive SMC (ASMC) techniques [15,16,17] are proposed to handle model uncertainties, disturbances, and actuator faults. These studies show some acceptable results, but they cannot tolerate the fault in the case of complete failure [18,19]. Some control methods are investigated to overcome the complete failure issue. Merheb et al. suggested the reconfiguration technique based on control allocation to transform a quadcopter into a tricopter [18]. The results can tolerate complete failure and demonstrate a good tracking performance. However, this method cannot detect the location of the fault and it requires an extra weight mounted on the opposite motor. There is another approach, based on backstepping [19], to transform the quadcopter into a birotor for emergency landing, but this method does not consider fault detection. In [20], a periodic solution combined with removing yaw motion was proposed to address one, two, or three propeller failures but this approach cannot use a fault detection scheme. Moreover, this work may not work in outdoor environments because GPS systems have a poor signal when removing yaw motion. It can be demonstrated that complete failure in one motor of a quadcopter requires removing yaw motion to ensure the controllability of the system because the quadcopter lacks redundancy in a dynamic model [11,20].

A hexacopter consists of six motors, including three motors rotating clockwise and three motors rotating counterclockwise. It was developed to increase the possibility of controllability of one or more actuator failures through its allocation matrix. Recent years have witnessed several works on the FTC problem of a hexacopter in the case of a motor failure. An attainable control set was proposed to evaluate the controllability of a hexacopter or an octocopter [21]. The optimal solution of thrust and torques is arrived at using parametric programming, which is obtained from the search tree method. In [22], an incremental backstepping fault-tolerant control is proposed for a hexacopter under an unknown control degradation occurring in the propulsion system. In [23], a Time Delay Control (TDC) method is presented to address single or multiple failure in the actuator. However, most of the above studies are tested and validated by simulation. A recent study presented experimental work on the FTC of a hexacopter using parametric programming formulation [24]. This work shows some good results in hovering or tracking performance after faults occur in one or two motors, but this design assumes that the location of motor failures is known to design the FTC approach. Although current studies proposed some good results on FTC of hexacopters, they lack experimental works or lack the fault detection unit to determine the location of a fault before designing the FTC method.

### 1.2. Main Contributions

In this paper, a fault-tolerant control approach is proposed for a hexacopter UAV to address one or two actuator faults with disturbances. Differing from previous studies, this article aims to integrate a fault detection unit into fault-tolerant control as a single unit. In detail, the fault detection unit is first used to detect the location of faults. Next, using this fault detection information, the motor failures are isolated and then a fault-tolerant control method is developed to hover the hexacopter through the reconfiguration technique.

This paper is organized as follows. Section 2 describes the dynamic model of the hexacopter. The attitude and altitude controller design are shown in Section 3. Section 4 presents the fault detection and isolation scheme. The experimental results are presented in Section 5 to validate the effectiveness of the proposed method. Section 6 presents the conclusions and the possibilities for future research.

## 2. Mathematical Model of a Hexacopter

**Figure 1:**
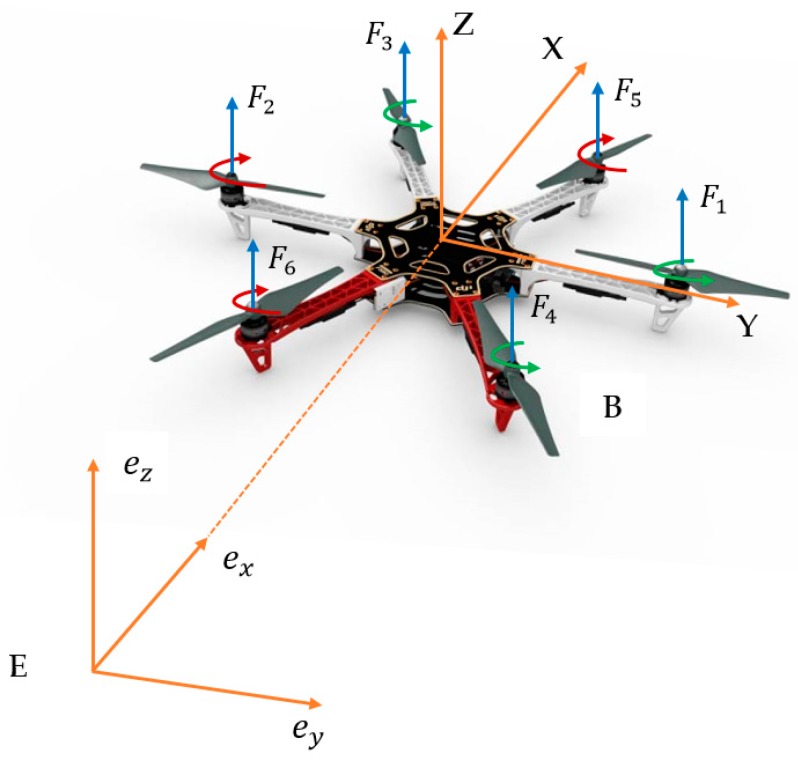
Hexacopter in the body and inertia frame.

Let us examine the body frame B and inertial frame E to describe a hexacopter dynamic (see Figure 1). For the body frame, the X−Y plane is placed at the surface while the Z-axis is denoted by the right hand rule. The center of gravity (CoG) of the hexacopter is placed at the origin of the body frame. The body frame is transformed to inertia frame using rotation matrix in Equation (1).

(1) $$R(\varphi ,\theta ,\psi )=\left[\begin{array}{ccc} c\psi c\theta & c\psi s\theta s\varphi -s\psi c\varphi & c\psi s\theta c\varphi +s\psi c\varphi \\ s\psi c\theta & s\psi s\theta s\varphi +c\psi c\varphi & s\psi s\theta c\varphi -s\varphi c\psi \\ -s\theta & c\theta s\psi & c\theta c\varphi \end{array}\right],$$

where s denotes sin, c denotes cos, and φ,θ,ψ stand for Euler angles.

The hexacopter shown in Figure 1 includes three motors (1, 3, 4) rotating counterclockwise and three motors rotating clockwise. Four control inputs $U_{1},\;\;U_{2},\;\;U_{3},$ and $U_{4}$ are defined as

(2) $$\left\{ \begin{array}{l} U_{1}=F_{1}+F_{2}+F_{3}+F_{4}+F_{5}+F_{6}\\ U_{2}=\left(F_{2}-F_{1}+(F_{3}+F_{6}-F_{4}-F_{5})/2\right)L\\ U_{3}=(F_{3}+F_{5}-F_{4}-F_{6})L\sqrt{3}/2\\ U_{4}=\tau_{2}+\tau_{5}+\tau_{6}-\tau_{1}-\tau_{3}-\tau_{4} \end{array}\right.,$$

where L is the arm length; $\tau_{i}=d\Omega_{i}^{2}$ and $F_{i}=b\Omega_{i}^{2}$ are the torques and forces generated from ith motor; $\Omega_{i}$ is the rotation speed; $U_{1}$ is the total thrust; $U_{2},\;\;U_{3},\;\;U_{4}$ are the torques in the directions φ,θ,ψ.

According to the Newton–Euler equation, the body dynamics is shown as [25]:

(3) $$\left\{ \begin{array}{l} m\ddot{r}=R\left[\begin{array}{l} 0\\ 0\\ U_{1} \end{array}\right]-\left[\begin{array}{l} 0\\ 0\\ mg \end{array}\right]-\dot{\omega }\times m\ddot{r}\\ I\ddot{\omega }=\left[\begin{array}{l} U_{2}\\ U_{3}\\ U_{4} \end{array}\right]-\dot{\omega }\times I\ddot{\omega } \end{array}\right.,$$

where m is the mass of the hexacopter; ω is the angular velocity vector; g is the gravity; I is the inertia vector; r is the position in inertial frame;

Finally, the orientation and translation motion of the hexacopter can be derived as [26]

(4) $$\left\{ \begin{array}{l} \ddot{x}=\left\{U_{1}(\cos\varphi sin\theta \cos\psi +\sin\varphi \sin\psi )-K_{x}\dot{x}\right\}/m\\ \ddot{y}=\left\{U_{1}(\cos\varphi sin\theta sin\psi -\sin\varphi \sin\psi )-K_{y}\dot{y}\right\}/m\\ \ddot{z}=-g+\left\{U_{1}(\cos\varphi cos\theta )-K_{z}\dot{z}\right\}/m\\ \ddot{\varphi }=\left(U_{2}+(I_{y}-I_{z})\dot{\theta }\dot{\psi }-J_{T}\dot{\theta }\Omega -K_{\varphi }\dot{\varphi }\right)/I_{x}\\ \ddot{\theta }=\left(U_{3}+(I_{z}-I_{x})\dot{\varphi }\dot{\psi }-J_{T}\dot{\varphi }\Omega -K_{\theta }\dot{\theta }\right)/I_{y}\\ \ddot{\psi }=\left(U_{4}+(I_{x}-I_{y})\dot{\varphi }\dot{\theta }-K_{\psi }\dot{\psi }\right)/I_{z} \end{array}\right.,$$

where Ω is the disturbance: $\Omega =\Omega_{3}+\Omega_{4}-\Omega_{1}-\Omega_{2}$; $U_{1}$ is the total thrust; $U_{2},\;\;U_{3},\;\;U_{4}$ are the torques in the directions φ,θ,ψ; $I_{x},\;\;I_{y},\;\;I_{z}$ are the moments of inertia of the hexacopter along the x,  y,  z axis; $K_{\varphi },\;\;K_{\theta },\;\;K_{\psi },\;K_{x},\;K_{y}$ represent drag coefficients; $J_{T}$ is the inertia of each rotor.

From Equation (4), the relationship between control variables and forces can be described by the allocation matrix T as:

(5) $$T=\left[\begin{array}{cccccc} 1 & 1 & 1 & 1 & 1 & 1\\ -L & L & L/2 & -L/2 & -L/2 & L/2\\ 0 & 0 & L\sqrt{3}/2 & -L\sqrt{3}/2 & L\sqrt{3}/2 & -L\sqrt{3}/2\\ -d/b & d/b & -d/b & -d/b & d/b & d/b \end{array}\right].$$

## 3. Attitude and Altitude Controller Design

### 3.1. Attitude Controller Design

**Figure 2:**
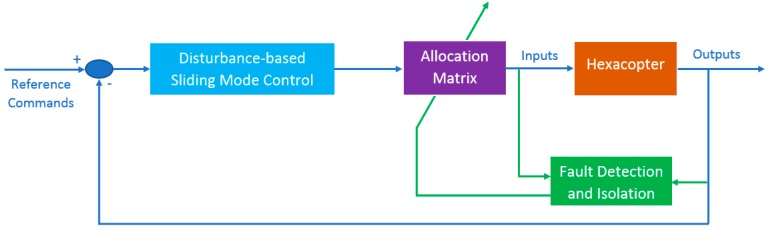
Control structure of a hexacopter**.**

The full control structure is shown in Figure 2. In this scheme, the disturbance-based sliding mode control method is proposed for attitude control and to handle disturbances, whereas the fault detection and isolation unit is used to detect fault occurrence. When the residuals from the fault detection unit exceed the threshold, the motor fault is isolated and then the allocation matrix is modified correspondingly to adopt the new geometry of the hexacopter.

Define $x={\left[\begin{array}{cccccc} \varphi & \dot{\varphi } & \theta & \dot{\theta } & \psi & \dot{\psi } \end{array}\right]}^{T}\;\;\;={\left[\begin{array}{cccccc} x_{1} & x_{2} & x_{3} & x_{4} & x_{5} & x_{6} \end{array}\right]}^{T}$ and $U={\left[\begin{array}{ccc} U_{2} & U_{3} & U_{4} \end{array}\right]}^{T}={\left[\begin{array}{ccc} u_{1} & u_{2} & u_{3} \end{array}\right]}^{T}$ as the state vector and control input vector, respectively. From Equation (4), each rotational movement equation has the following form:

(6) $$\left\{ \begin{array}{l} {\dot{x}}_{2i-1}=x_{2i}\\ {\dot{x}}_{2i}=f_{i}(x)+h_{i}u_{i}(t)+d_{i}(x,t) \end{array}\right.$$

The desired attitude is denoted as $x_{i}^{d}$. The control goal is to generate the control signal $u_{i}$ such that the hexacopter can track the desired attitude, i.e., $x_{2i-1}(t)\to x_{i}^{d}$ as t→∞, i=1, 2, 3.

**Assumption** **1.** The disturbance is assumed to be bounded and satisfied $\left|d_{i}\right|\leq D_{i}$.

**Assumption** **2.** The disturbance changes slowly and satisfies ${\dot{d}}_{i}=0$.

**Assumption** **3.** The estimation error of disturbance is bounded by $\left|{\dot{\tilde{d}}}_{i}\right|\leq \Gamma_{i}$.

According to [27], a nonlinear disturbance-based sliding mode control is proposed by

(7) $$\left\{ \begin{array}{l} {\hat{d}}_{i}=z_{i}+\delta_{i}x_{2i}\\ {\dot{z}}_{i}=-\delta_{i}z_{i}-\delta_{i}(\delta_{i}x_{2i}+f_{i}(x)+g_{i}(x)u_{i}) \end{array}\right.,$$

where ${\hat{d}}_{i}$ is the estimate of $d_{i}$, $z_{i}$ is the observation auxiliary vector, $\delta_{i}>0$ is the observer gain. The estimation error of disturbance is denoted as:

(8) $${\tilde{d}}_{i}=d_{i}-{\hat{d}}_{i}.$$

From Assumption 2, the derivative of disturbance estimation error can be defined as:

(9) $$\begin{array}{ll} {\dot{\tilde{d}}}_{i} & ={\dot{d}}_{i}-{\dot{\hat{d}}}_{i}\\ & =-{\dot{z}}_{i}-\delta_{i}{\dot{x}}_{2i}\\ & =\delta_{i}({\hat{d}}_{i}-\delta_{i}{\dot{x}}_{2i})+\delta_{i}(\delta_{i}x_{2i}+f_{i}(x)+g_{i}(x)u_{i})-\delta_{i}(f_{i}(x)+g_{i}(x)u_{i}+d_{i})\\ & =-\delta_{i}{\tilde{d}}_{i} \end{array}$$

Denote the control error:

(10) $$e_{i}=x_{2i-1}-x_{i}^{d}\;.$$

The sliding surface is expressed by:

(11) $$s_{i}={\dot{e}}_{i}+k_{i}e_{i}\;,$$

where $k_{i}$ is the positive parameter.

Taking the derivative of sliding surface

(12) $${\dot{s}}_{i}={\ddot{e}}_{i}+k_{i}{\dot{e}}_{i}\;.$$

From Equations (7)–(9), ${\dot{s}}_{i}$ can be derived as

(13) $${\dot{s}}_{i}=f_{i}(x)+h_{i}u_{i}(t)+d_{i}(x,t)-{\ddot{x}}_{i}^{d}+c_{i}{\dot{e}}_{i}\;.$$

**Theorem** **1.** *Consider the sliding surface is designed by Equation (11). Suppose that the following control law is implemented by*

(14) $$u_{i}=(-f_{i}(x,t)-{\hat{d}}_{i}(x,t)+{\ddot{x}}_{i}^{d}-c_{i}{\dot{e}}_{i}-\Gamma_{i}\mathrm{sign}(s_{i})/h_{i}\;.$$

Then the nonlinear system (Equation (6)) is stable and the control errors are forced to zero.

**Proof of Theorem** **1.** Choose the Lyapunov function as follows [26]

(15) $$V=\frac{1}{2}s_{i}^{2}+\frac{1}{2}{\dot{\tilde{d}}}_{i}^{2}\;.$$

Take the first derivative of the Lyapunov function

(16) $$\begin{array}{l} \dot{V}=s_{i}{\dot{s}}_{i}-\delta_{i}{\tilde{d}}_{i}^{2}\\ \;\;\;\;\;\;=s_{i}({\tilde{d}}_{i}-\Gamma_{i}\mathrm{sign}(s_{i}))-\delta_{i}{\tilde{d}}_{i}^{2}\\ \;\;\;\;\;\;\leq -\left(\Gamma_{i}-\left|{\tilde{d}}_{i}\right|\right)\left|s_{i}\right|-\delta_{i}{\tilde{d}}_{i}^{2}\\ \;\;\;\;\;\;\leq 0 \end{array}$$

Therefore, according to the control strategy (Equation (14)) and disturbance observer (Equation (9)), the closed-loop system can maintain stability when both actuator faults and external disturbances occur. ☐

**Remark** **1.** With the proposed disturbance observer-based sliding mode control, the discontinuous control gain is decreased, and it is required to be larger than the bound of estimation error rather than that of disturbance.

**Remark** **2.** *To handle the chattering issue due to the sign function from Equation (14), a saturation function is denoted as*

(17) $$sat(s_{i})=\left\{ \begin{array}{l} s_{i}\;\;\;\;\;\;\;\;\;\;\;\;\;\;\;\;\;\;\;\mathrm{if}\;\left|s_{i}\right|\leq 1\\ sign(s_{i})\;\;\;\;\;\;\;\mathrm{if}\;\left|s_{i}\right|>1 \end{array}\right.,\;\;\;i=1,\;2,\;3.\;$$

### 3.2. Altitude Controller Design

A PID controller is applied to control the vertical movement of the hexacopter as follows

(18) $$\ddot{Z}={\ddot{Z}}_{d}+K_{dz}(\dot{Z}-{\dot{Z}}_{d})+K_{pz}(Z-Z_{d})+K_{iz}\int (Z-Z_{d})dt,\;$$

where $\;Z_{d}$ are the desired altitude in the  z directions, respectively;  Z is actual value; and $K_{pz},\;\;K_{dz},$ and $K_{iz}$ are the controller gains.

## 4. Fault Detection and Fault-Tolerant Control System

Fault detection consists of two modules: residual generation and residual validation. Residual generation is defined as the difference between system output and state observer. The information of residual generation is used for the residual validation module that determines fault-free or faulty case. When the output of residual validation module is in the faulty case, the fault isolation (FI) module is activated. The obtained FI information is used to determine the location of the fault, which is based on the magnitude and sign of residual validation module. Finally, the fault-tolerant control system aims to change the allocation matrix to hover and land the hexacopter.

### 4.1. Residual Generation

A nonlinear observer presented in [28] is applied for fault detection and isolation (FDI). Let us consider the following nonlinear model:

(19) $$\left\{ \begin{array}{l} \dot{x}(t)=Ax(t)+BL_{1}u(t)+h(x(t),L_{1}u(t))\\ y(t)=Cx(t) \end{array}\right.,$$

where $x(t)=\left[\begin{array}{cccccccc} \varphi & \dot{\varphi } & \theta & \dot{\theta } & \psi & \dot{\psi } & z & \dot{z} \end{array}\right]$, $u(t)=\left[\begin{array}{cccc} U_{2} & U_{3} & U_{4} & U_{1} \end{array}\right]$, y(t) are the state vector, control input vector, and the output vector; A,  B,  C are the system matrices; $L_{1}$ is the fault matrix; $h(x(t),L_{1}u(t))$ is the nonlinear function.

From the state model in Equation (19), the following conditions are designed for a Thau observer [28]:

**Condition** **1.** the pair (C, A) is observable.

**Condition** **2.** the nonlinear part h(x(t),u(t)) is Lipschitz and differentiable continuously with a constant γ, i.e., $\left\|h(x_{1}(t),u(t))-h(x_{2}(t),u(t))\right\|\leq \gamma \left\|x_{1}-x_{2}\right\|$.

If all conditions are satisfied, the Thau observer can be derived as [28]:

(20) $$\left\{ \begin{array}{l} \dot{\hat{x}}(t)=A\hat{x}(t)+Bu(t)+K(\hat{y}(t)-y(t))+h(\hat{x}(t),u(t))\\ \hat{y}(t)=C\hat{x}(t) \end{array}\right.,$$

where $\hat{x}(t)=\left[\begin{array}{cccccccc} \hat{\phi } & \dot{\hat{\phi }} & \hat{\theta } & \dot{\hat{\theta }} & \hat{\psi } & \dot{\hat{\psi }} & \hat{z} & \dot{\hat{z}} \end{array}\right]$ is the state observer vector; $\hat{y}(t)$ is the observer output vector; K is the designed matrix and determined by

(21) $$K=P_{\varepsilon }^{-1}C^{T}.$$

The matrix $P_{\varepsilon }$ is achieved from the following equation

(22) $$A^{T}P_{\varepsilon }+P_{\varepsilon }A-C^{T}C+\varepsilon C^{T}P_{\varepsilon }=0,$$

where ε is a positive value such that $P_{\varepsilon }\geq 0$.

The residuals are defined as the deviation between real output of system and state output:

(23) $$\begin{array}{l} r_{1}=\varphi -\hat{\varphi },\;\;\;r_{2}=\theta -\hat{\theta }\\ r_{3}=\psi -\hat{\psi },\;\;r_{4}=z-\hat{z}\\ r_{5}=\dot{\varphi }-\dot{\hat{\varphi }},\;\;r_{6}=\dot{\theta }-\dot{\hat{\theta }}\\ r_{7}=\dot{\psi }-\dot{\hat{\psi }},\;\;r_{8}=z-\dot{\hat{z}} \end{array}$$

### 4.2. Residual Validation

The residual validation module is used to detect the fault-free or faulty case of system, which is compared with upper or lower thresholds. The decision logic is determined as follows:

$$\left\{ \begin{array}{l} r_{i}(t)\geq H_{i}\;\;\mathrm{or}\;\;r_{i}\leq M_{i}\;\;\;\text{in fault-free case}\\ r_{i}(t)<H_{i}\;\;\mathrm{and}\;\;r_{i}(t)>M_{i}\;\;\text{in faulty case} \end{array}\right.$$.

Using this residual validation module, the fault isolation and fault-tolerant control methods will be designed in Section 4.3 to isolate the fault and to reconfigure the allocation matrix.

### 4.3. Fault Isolation and Fault-Tolerant Control

When residual validation of FD system detects the faulty case, the FI module is activated. To determine the location of the fault, the knowledge of residuals $r_{5},\;\;r_{6},\;\;r_{7},\;r_{8}$ should be used. The failure motor is isolated through the sign and magnitude of these residuals. The map of failure in one or two motors can be concluded in Table 1 and Table 2, which depends on the sign and magnitude of residuals. In these Tables, the “Controllable” column means that the hexacopter can hover its position and make a landing mode during flight test. After the fault is isolated from the FI module, the allocation matrix is reconfigured correspondingly for fault accommodation. 

## 5. Experimental Results

### 5.1. Experimental Setup

**Figure 3:**
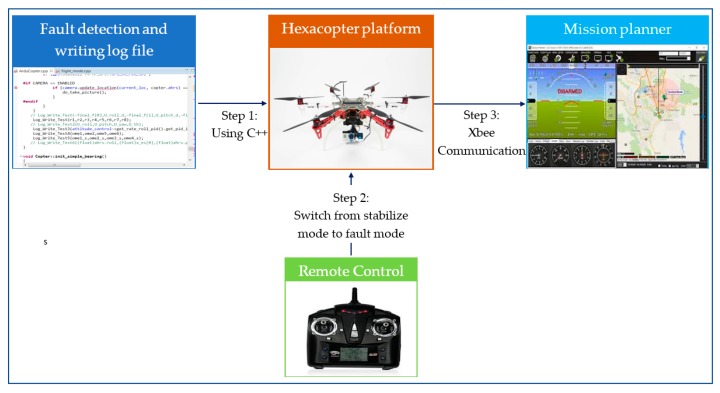
Procedure of the experimental test.

The fault-tolerant control, fault detection, and fault isolation methods in Section 3 and Section 4 were tested on a DJI F550 platform [29]. These algorithms were developed on Pixhawk2 flight controller [30], which requires the C++ program for implementation [31]. The firmware version 3.5 was chosen for the flight controller. During testing, the faults were injected by limiting the pulse width modulation (PWM) [32] through a remote control that can switch to faulty mode from stabilize mode. The flight data were monitored using Mission Planner (MP) [33] software through Xbee wireless communication [34]. The fault detection data were achieved through a log file that is coded in C++ by the user. The experimental procedure is summarized in Figure 3.

### 5.2. Results

To demonstrate the performance of the proposed control scheme, different simulation tests on a hexacopter are considered. The hexacopter parameters are shown in Table 3. 

The parameters of disturbance-based sliding mode control are chosen as: $k_{i}=10,\;\;\delta_{i}=3,\;\;i=1,2,3$. The parameters of fault detection scheme are chosen as: $M_{i}=-0.35$ and $H_{i}=0.35$. $A=\left[\begin{array}{cc} 0_{4\times 4} & I_{4\times 4}\\ 0_{4\times 4} & 0_{4\times 4} \end{array}\right]\;,$$K=\left[\begin{array}{cccc} 1.1 & 0 & 0 & 0\\ 0 & 1.1 & 0 & 0\\ 0 & 0 & 1.1 & 0\\ 0 & 0 & 0 & 1.1\\ 0.3025 & 0 & 0 & 0\\ 0 & 0.3025 & 0 & 0\\ 0 & 0 & 0.3025 & 0\\ 0 & 0 & 0 & 0.3025 \end{array}\right]$, $C=\left[\begin{array}{cccccccc} 1 & 0 & 0 & 0 & 0 & 0 & 0 & 0\\ 0 & 1 & 0 & 0 & 0 & 0 & 0 & 0\\ 0 & 0 & 1 & 0 & 0 & 0 & 0 & 0\\ 0 & 0 & 0 & 1 & 0 & 0 & 0 & 0 \end{array}\right]$, $h(x,u)=\left[\begin{array}{c} 0\\ 0\\ 0\\ 0\\ (\dot{\theta }\dot{\psi }(I_{y}-I_{z})-J_{T}\dot{\theta }\Omega )/I_{x}\\ (\dot{\varphi }\dot{\psi }(I_{z}-I_{x})-J_{T}\dot{\varphi }\Omega )/I_{y}\\ \dot{\varphi }\dot{\theta }(I_{x}-I_{y})/I_{z}\\ -g \end{array}\right]$$B=\left[\begin{array}{cccc} 0 & 0 & 0 & 0\\ 0 & 0 & 0 & 0\\ 0 & 0 & 0 & 0\\ 0 & 0 & 0 & 0\\ 0 & -L/I_{x} & 0 & L/I_{x}\\ -L/I_{y} & 0 & L/I_{y} & 0\\ d/(bI_{z}) & -d/(bI_{z}) & d/(bI_{z}) & -d/(bI_{z})\\ 1/m & 1/m & 1/m & 1/m \end{array}\right]$.

In scenario 1, partial loss effectiveness in actuator 2 is presented to verify the disturbance-based sliding mode control and residuals from Table 1. To show the fault detection and isolation effectiveness, scenarios 2, 3, and 4 show the case of one motor failure, two motor failure in sequence, or two motor failure simultaneously.

#### 5.2.1. Partial Loss in Actuator 2

**Figure 4:**
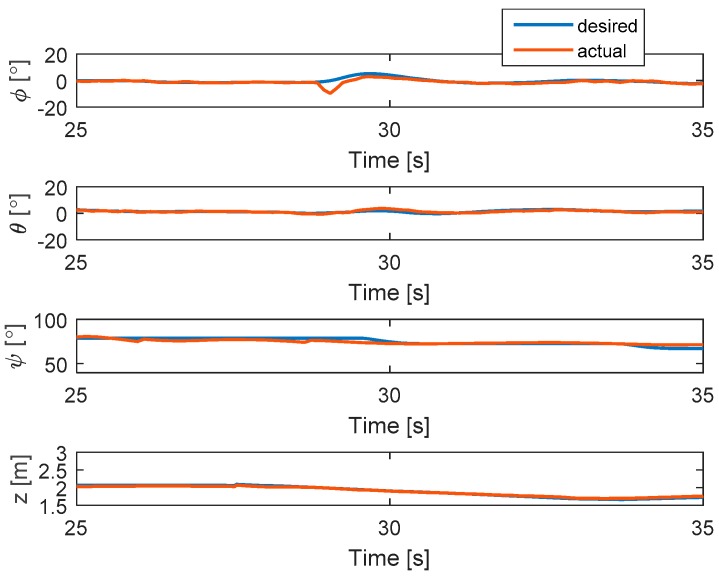
States of the system.

A 30% loss of control effectiveness in the actuator fault 2 is simulated. The hexacopter can hover at the altitude of 2 m and the fault occurs at t = 28 s. It can be seen in Figure 4 that the Euler angles can converge quickly to the desired angles after a fault occurrence due to the disturbance observer-based controller. Moreover, the actual altitude can also track the desired one after the fault occurrence. 

**Figure 5:**
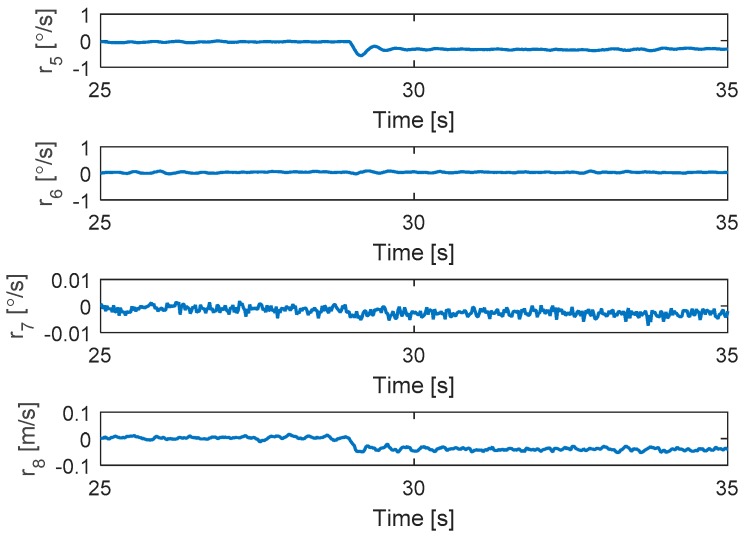
Residuals generated from fault detection schemes.

The residuals are shown in Figure 5. Interestingly, the residuals are the same as those in the fault map presented in Table 1. Since the residuals do not exceed the thresholds, the attitude still uses the disturbance-based sliding mode control for accommodation without changing the allocation matrix.

**Figure 6:**
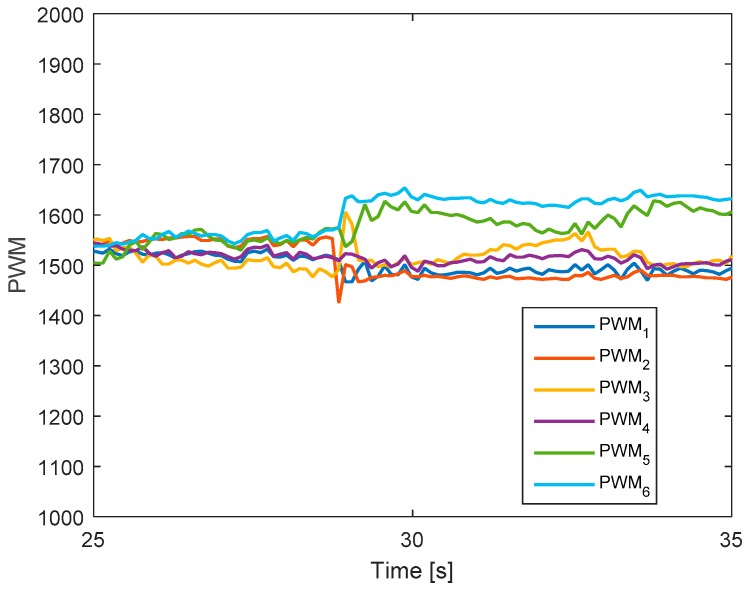
Output signals of the system.

The output signals are illustrated in Figure 6. It should be noted that before the fault (up to t = 28 s), the six PWM signals are similar. After fault is injected in motor 2, the second PWM is decreased and then its value increases again to maintain the position of the hexacopter. 

The experimental work can be found at https://www.youtube.com/watch?v=en-h_HwVh0k or in the Supplementary Materials.

#### 5.2.2. Complete Loss in Actuator 4

**Figure 7:**
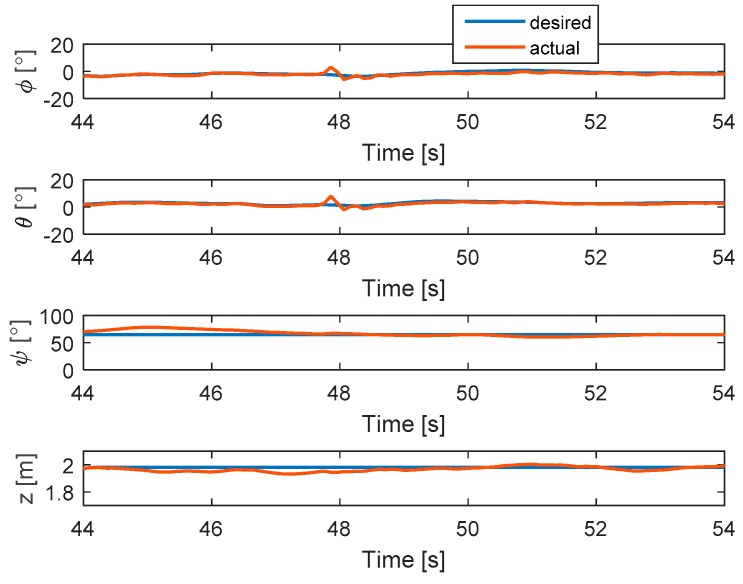
States of the system.

**Figure 8:**
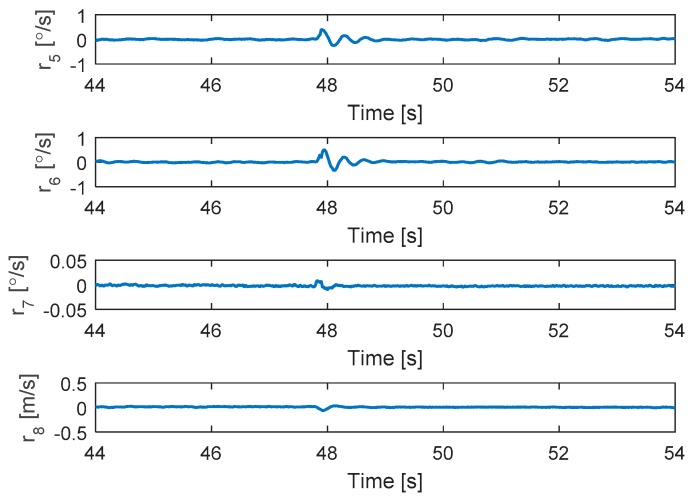
Residuals generated from fault detection schemes.

In this test, complete failure is introduced to motor 4. The hexacopter is made to hover at the height of 2 m and the fault is injected at t = 47.8 s. As can be seen in Figure 7, the attitude angles can track the desired angles although the they have some small oscillations after a fault occurs. The residuals of system are presented in Figure 8. It is shown that when fault occurs, all signs of residuals are similar to those in Table 1 at the beginning and then they go back to the zero. Unlike previous case, the residuals here converge to the original point because the magnitude of residuals exceed the threshold and then the motor 4 is isolated after fault occurrence. 

**Figure 9:**
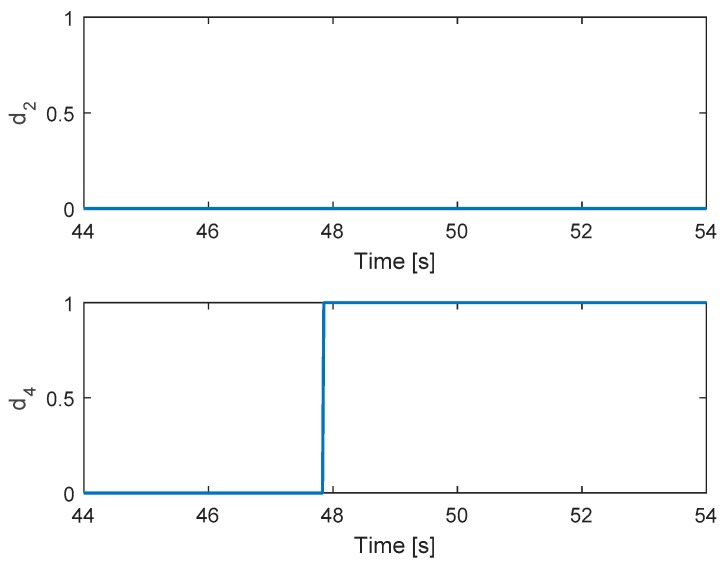
Fault detection of actuators 2 and 4.

**Figure 10:**
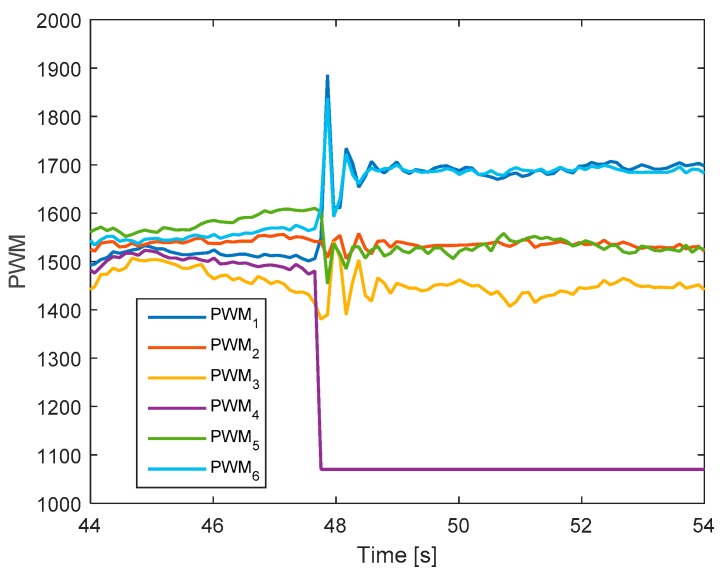
Output signals of the system.

The detection unit of motors 2 and 4 is presented in Figure 9. It is clear that only motor 4 is detected from the FD unit. Figure 10 shows the corresponding output signals. It shows that the PWM of motor 4 has a minimum value due to fault injection. The other PWMs are changed correspondingly to maintain the position of hexacopter because the motor 4 is isolated from the allocation matrix.

The experimental work can be found at https://www.youtube.com/watch?v=rSSoVS4nDaE or in the Supplementary Materials.

#### 5.2.3. Complete Loss of Two Motors in Sequence

**Figure 11:**
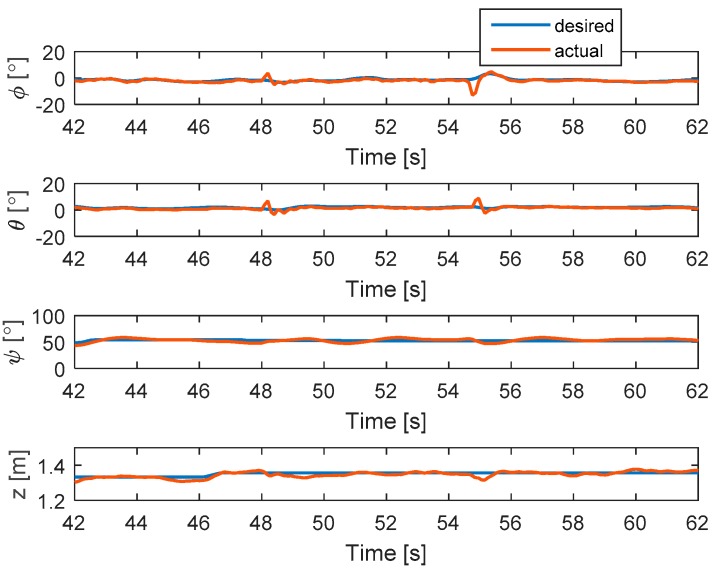
States of the system.

**Figure 12:**
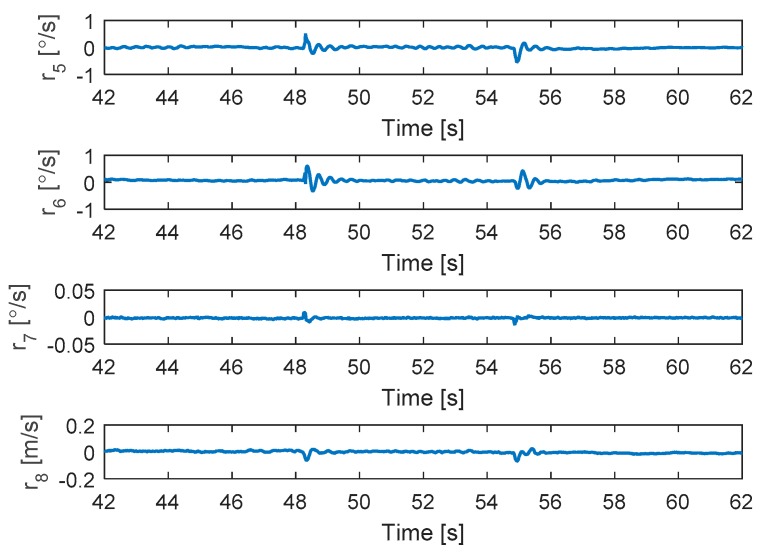
Residuals generated from fault detection schemes.

In this test, the faults are injected into motors 4 and 2 at 48 and 54.5 s, respectively. The hexacopter is made to hover at the height of 1.35 m. As can be seen in Figure 11, the attitude angles can track the desired angles although the they have some small oscillations after faults occur in motors 4 and 2. The residuals of system are presented in Figure 12. Unlike the case in Section 5.2.2, after the fault occurs in motor 4, the system can still detect and isolate the fault of motor 2. 

**Figure 13:**
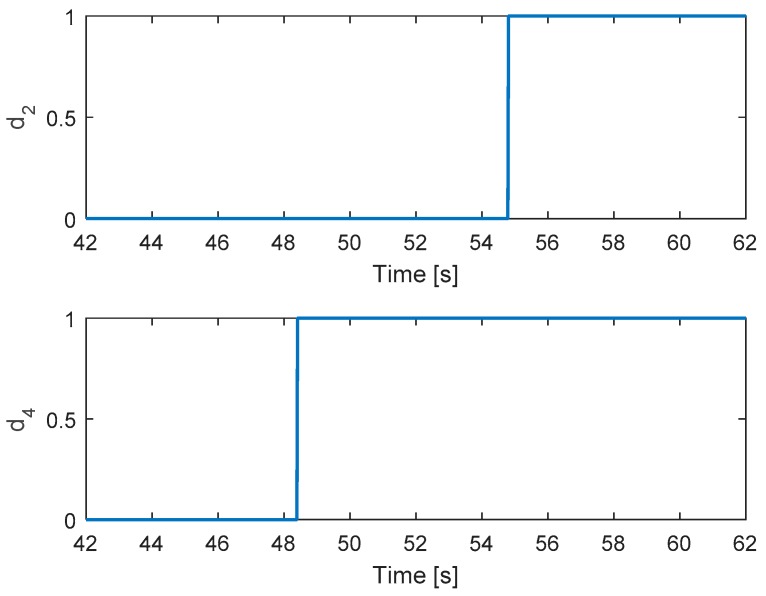
Fault detection of actuators 2 and 4.

**Figure 14:**
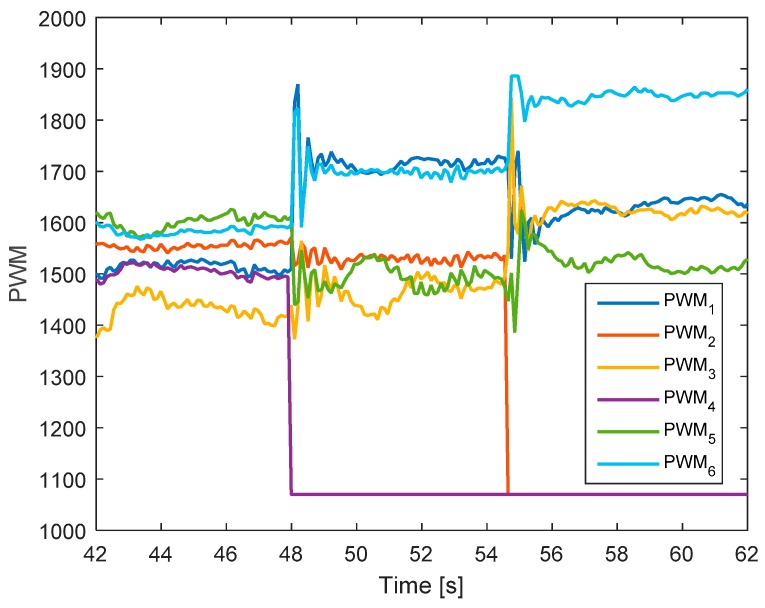
Output signals of the system.

Figure 13 shows the detection unit of the system. It is clear that the faults in motors 4 and 2 are detected from the FD unit because their residuals’ magnitudes exceed the threshold values. The corresponding output signals are presented in Figure 14. It shows that the PWMs of motors 4 and 2 have minimum values due to fault injections. The other PWMs are changed correspondingly to maintain the position of the hexacopter because the motors 4 and 2 are isolated from the allocation matrix.

The experimental work can be found at https://www.youtube.com/watch?v=yv-L4qBZ8EU or in the Supplementary Materials.

#### 5.2.4. Complete Loss in Two Motors at the Same Time

**Figure 15:**
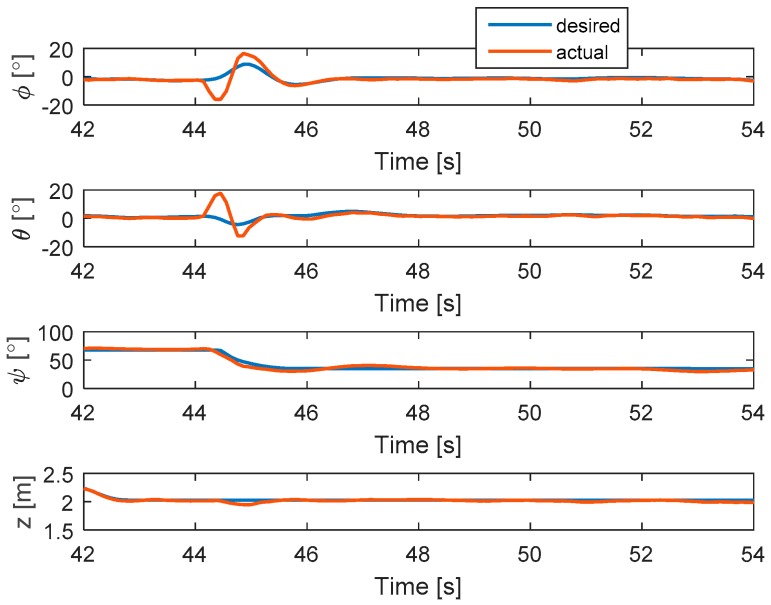
States of the system.

**Figure 16:**
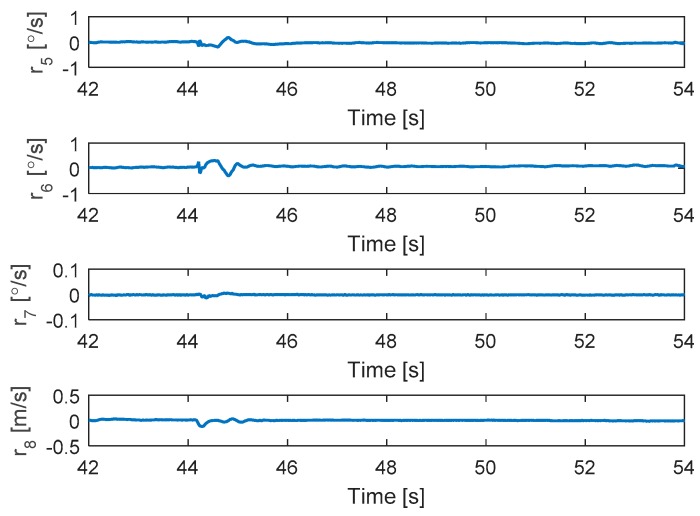
Residuals generated from fault detection schemes.

In this scenario, the faults are injected to motors 2 and 4 at the same time. The hexacopter is made to hover at the height of 2 m and the fault occur at 44 s. As can be seen in Figure 15, the attitude angles can track the desired angles although the they have some oscillations after faults occur in motors 4 and 2. The residuals of system are presented in Figure 16. It is shown that when a fault occurs, all signs of the residuals are similar to those in Table 1 at the beginning and then they converge to zero. This convergence is because the residuals exceed the threshold values and two motor failures are isolated.

**Figure 17:**
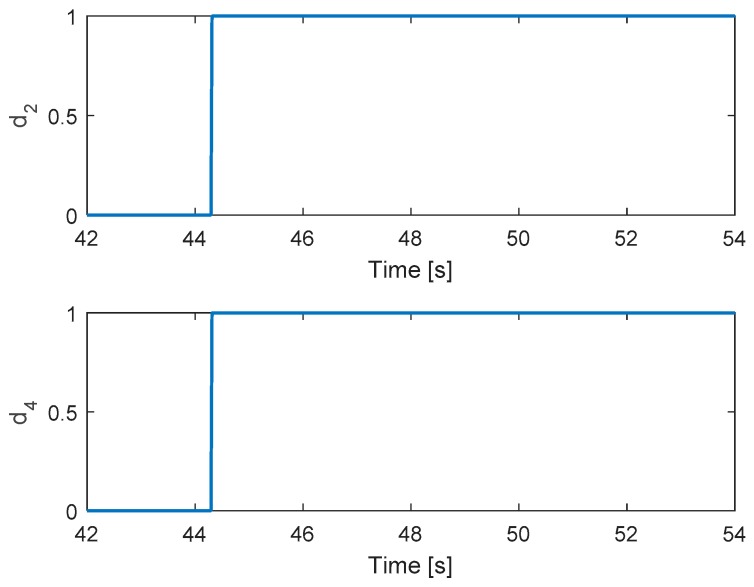
Fault detection of actuators 2 and 4.

**Figure 18:**
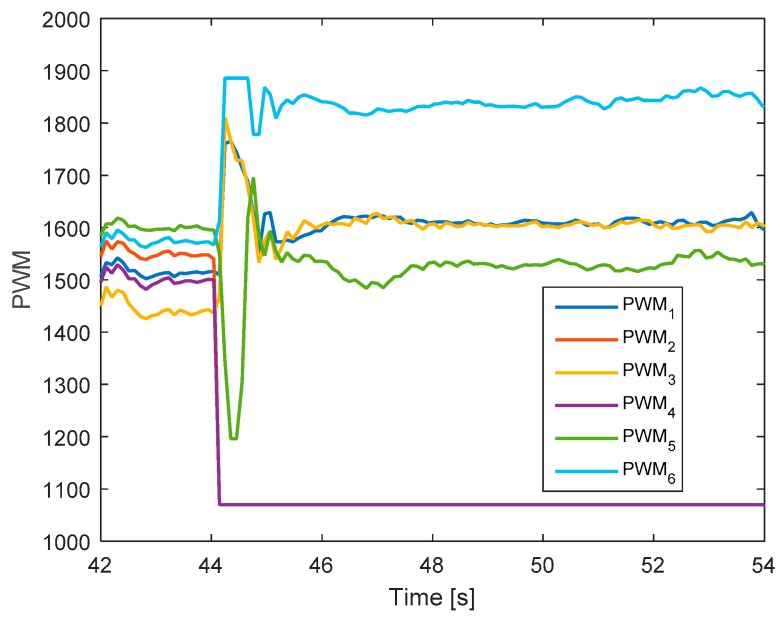
Output signals of the system.

Figure 17 shows the detection unit of system. It is clear that the faults in motors 4 and 2 are detected from the FD unit at the same time because their residuals’ magnitudes exceed the threshold values. Figure 18 shows the corresponding output signals. It shows that the PWMs of motors 4 and 2 have minimum values due to fault injections. The other PWMs are changed correspondingly to maintain the position of the hexacopter because motors 4 and 2 are isolated from the allocation matrix.

The experimental work can be found at https://www.youtube.com/watch?v=uZuG44NqL8s or in the Supplementary Materials.

**Remark** **3.** In this research, two motor failures are presented. It should be mentioned that the total thrust generated from remaining motors should be larger than the gravity force of the hexacopter. Therefore, users should choose suitable motors and hexacopter masses for safety purposes.

**Remark** **4.** *Some previous studies assume that the location of fault is known, and they only focus on fault-tolerant control design to handle fault. In comparison with previous studies, our research can maintain the hexacopter up to two motor failures in presence of disturbances with two scenarios: sequential faults and simultaneous faults. We believe that our research could be innovative in commercial software of the Pixhawk2 flight controller.*

**Remark** **5.** In several flight tests on the Pixhawk2 flight controller, the hexacopter crashed because it lacks the fault detection and fault-tolerant control algorithm. Therefore, it is difficult to gather data of the Pixhawk2 flight controller for comparison.

**Remark** **6.** Consider the frames that have more than six motors, the fault detection and fault-tolerant control schemes are more complex, which is out of scope of this research and will be investigated in further research.

## 6. Conclusions

In this paper, the dynamic model of a hexacopter was explored with regard to disturbances and actuator faults. A fault detection based on a Thau observer was applied to monitor the location of one or two rotor failures. When the residuals exceeded the threshold values, the motor failure was isolated and then allocation matrix was changed. The results show that the proposed fault detection and isolation can be used to reconfigure the geometry of a hexacopter, hence making a safe flight when a fault occurs. Compared with previous studies, this research is innovative in several ways. First, the fault detection is integrated into fault-tolerant control as a single unit. Second, with the proposed disturbance observer-based sliding mode control, the discontinuous control gain is reduced, and it is required to be larger than the bound of estimation error rather than that of disturbance. Finally, the experimental works show that the hexacopter can handle complete fault in two cases: sequential fault and simultaneous fault. However, this study may not cover all cases of one motor or two motor failures. This limitation can be addressed by rotation of the coordinates or scarifying the yaw control. Future work should consider this problem and propose a suitable method for implementation.

## Figures and Tables

**Table 1:** Map of one motor failure.

Motor Failure	$r_{5}$	$r_{6}$	$r_{7}$	$r_{8}$	Controllable
$f_{1}$	+	0	+	−	Yes
$f_{2}$	−	0	−	−	Yes
$f_{3}$	−	−	+	−	No
$f_{4}$	+	+	+	−	Yes
$f_{5}$	+	−	−	−	No
$f_{6}$	−	+	−	−	Yes

Where $f_{i}$ is the fault occurring on motor i; the sign “−” denotes a normal negative value; the sign “+” denotes a normal positive value.

**Table 2:** Map of two motor failures.

Motor Failure	$r_{5}$	$r_{6}$	$r_{7}$	$r_{8}$	Controllable
$f_{12}$	0	0	0	−−	Yes
$f_{13}$	+	−	++	−−	No
$f_{14}$	+	+	++	−−	No
$f_{15}$	+	−	−	−−	No
$f_{16}$	+	+	−	−−	Yes
$f_{23}$	−−−	−	+	−−	No
$f_{24}$	−	+	+	−−	Yes
$f_{25}$	−	−	−−	−−	No
$f_{26}$	−−	+	−−	−−	No
$f_{34}$	−	−	++	−−	No
$f_{35}$	0	−	0	−−	No
$f_{36}$	−−	−	+	−−	No
$f_{45}$	+	−	−	−−	No
$f_{46}$	0	+	0	−−	No
$f_{56}$	+	+	−−	−−	No

Where $f_{ij}$ is the fault occurring on motor i  and  j, i<j; the signs “−−”, “−−−” denote “small negative value”, and “very small negative value”; the signs “++”, “+++” denote “big positive value”, and “very big positive value”.

**Table 3:** Hexacopter parameters.

Parameter	Description	Value
L	Arm length	0.275 m
b	Thrust coefficient	$1.177\times 10^{-5}$ N/m^2^
d	Drag coefficient	$1.855\times 10^{-7}$
m	Mass	2.5 kg
$I_{x};\;I_{y};\;I_{z}$	Moments of inertia	0.00915; 0.00915; 0.01187 kg·m^2^

## Supplementary Materials

The following are available online at https://www.mdpi.com/1424-8220/19/21/4721/s1.
